# A data-driven algorithm to support the clinical decision-making of patient extrication following a road traffic collision

**DOI:** 10.1186/s13049-023-01153-2

**Published:** 2023-12-04

**Authors:** Eyston Vaughan-Huxley, Joanne Griggs, Jasmit Mohindru, Malcolm Russell, Richard Lyon, Ewoud ter Avest

**Affiliations:** 1 Air Ambulance Kent Surrey Sussex, Hanger 10 Redhill Aerodrome, Redhill, RH1 5YP UK; 2 Kings College Hospital, Denmark Hill, London, SE5 9RS UK; 3 University of Surrey, Duke of Kent Building, Guildford, GU2 7XH UK; 4 Department of Emergency Medicine, University Medical Center Groningen, Groningen, The Netherlands

## Abstract

**Background:**

Some patients involved in a road traffic collision (RTC) are physically entrapped and extrication is required to provide critical interventions. This can be performed either in an expedited way, or in a more controlled manner. In this study we aimed to derive a data-driven extrication algorithm intended to be used as a decision-support tool by on scene emergency service providers to decide on the optimal method of patient extrication from the vehicle.

**Methods:**

A retrospective observational study was performed of all trauma patients trapped after an RTC who were attended by a Helicopter Emergency Medical Service (HEMS) in the United Kingdom between March 2013 and December 2021. Variables were identified that were associated with the need for HEMS interventions (as a surrogate for the need for expedited extrication), based on which a practical extrication algorithm was devised.

**Results:**

During the study period 12,931 patients were attended, of which 920 were physically trapped. Patients who scored an “A” on the AVPU score (n = 531) rarely required HEMS interventions (3%). Those who did were characterised by a shorter than average (29 vs. 37 min) 999/112 emergency call to HEMS on-scene arrival interval. A third of all patients responding to voice required HEMS interventions. Absence of a patent airway (OR 6.98 [1.74–28.03] *p* < .001) and the absence of palpable radial pulses (OR 9.99 [2.48–40.18] *p* < .001) were independently associated with the need for (one or more) HEMS interventions in this group. Patients only responding to pain and unresponsive patients almost invariably needed HEMS interventions post extrication (90% and 86% respectively). Based on these findings, a practical and easy to remember algorithm “APEX” was derived.

**Conclusion:**

A simple, data-driven algorithm, remembered by the acronym “APEX”, may help emergency service providers on scene to determine the preferred method of extrication for patients who are trapped after a road traffic collision. This has the potential to facilitate earlier recognition of a ‘sick’ critical patient trapped in an RTC, decrease entrapment and extrication time, and may contribute to an improved outcome for these patients.

**Supplementary Information:**

The online version contains supplementary material available at 10.1186/s13049-023-01153-2.

## Introduction

Road traffic collisions (RTCs) are a common dispatch reason for Helicopter Emergency Medical Services (HEMS) [1]. Sometimes patients involved in an RTC are trapped, either physically, or by the nature of their injuries [2–4]. Prolonged entrapment has been associated with an increased mortality [5, 6] and extrication is often mandatory before critical interventions can be provided.

Self-extrication has been recommended as the primary route of egress for patients following an RTC [7–9, 12, 13] and various algorithms have been devised to guide clinicians in the facilitation of this process [5–7]. However, sometimes self-extrication is not possible, and patients need assisted extrication by the emergency services due to the presence of entrapment, time-critical injuries, or both [4, 7]. In general, in these instances, a minimally invasive extrication approach is preferable [8, 9, 11, 14].

The extrication plan is always a balance between the need for critical interventions to be performed within a specific time frame, the need to provide spinal precautions and the risks for emergency personnel involved. Some patients will need expedited extrication to perform life-saving procedures such as pre-hospital emergency anaesthesia (PHEA), blood transfusion and surgical procedures such as thoracostomy and resuscitative thoracotomy post-extrication, whereas in other patients a more controlled extrication strategy is preferred to minimise the risk of spinal injuries, maximise clot stability and meet analgesic requirements for improved pain management [9].

To date, no guidance is available for the emergency medical services (EMS) on scene to inform decision making about the preferred extrication technique in patients who are mechanically trapped and cannot self-extricate [7, 10]. Unified guidance, to be used by all emergency services, could improve on-scene joint working. Therefore, in this study we aim to construct a data driven extrication algorithm generated from clinical findings in our patient cohort, as well as historical evidence that can be used as a decision-support tool by emergency service providers on scene to facilitate extrication decisions for mechanically trapped patients after an RTC.

## Methods

### Study design

A retrospective analysis was performed of all trauma patients trapped after an RTC who were attended by Air Ambulance Charity Kent Surrey Sussex (KSS) over an 8-year period from 1 March 2013 to 1 December 2021, in order to devise a data driven extrication algorithm based on easily obtainable patient and injury characteristics.

We investigated how patient and injury characteristics were related to the need to perform HEMS interventions on scene after extrication. HEMS interventions were chosen as an endpoint as due to their high acuity they reflect the need for expedited (as opposed to more controlled) extrication. Based on the identified predictive factors in combination with historical data, a practical algorithm was constructed.

### Study setting

KSS provides pre-hospital emergency medical cover to three counties (7200km^2^) in the southeast of England, with a resident population of 4.5 million and a transient population of 8-million. Two doctor-paramedic teams respond 24/7 in either a helicopter or a rapid response vehicle from one operational base, attending approximately 2000 patients per year. Tertiary trauma care in the region is offered at four major trauma centres (MTCs). KSS provides pre-hospital interventions alongside the regional Ambulance Trust and the Critical Care Paramedic (CCP) workforce.

### Dispatch model

KSS operates a dedicated dispatch desk which works in conjunction with the Critical Care Desk and is co-located at the regional Ambulance Trust. Prior to January 2016 the HEMS dispatch desk was led by HEMS Paramedics and subsequent to this, by a non-clinical HEMS Dispatcher, aided by a tasking algorithm based upon expert internal consensus [11]. Taskings are allocated according to mechanism of injury, patient clinical condition and geographical location. Entrapment following a RTC is amongst the mechanism-criteria to justify a rapid HEMS dispatch (Supplementary Material).

**Fig. 1:**
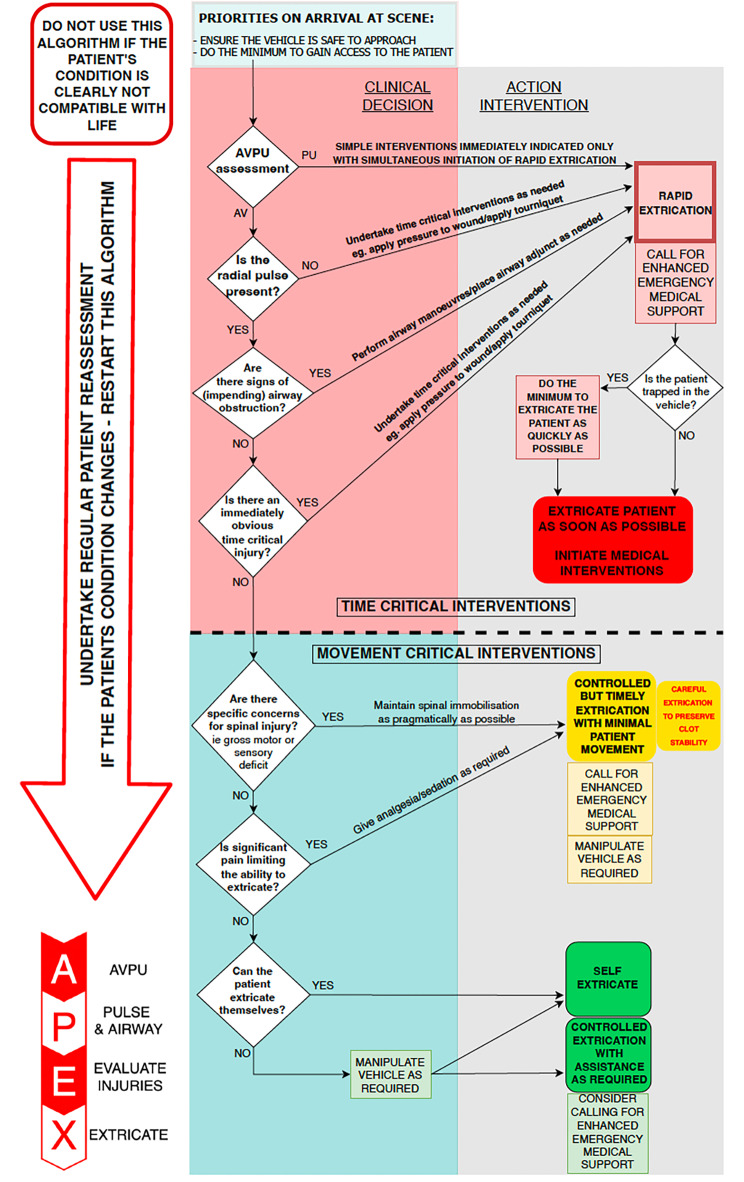
APEX Algorithm: A data driven proposed algorithm for clinical prioritisation of traumatically injured patients requiring extrication from a motor vehicle Proposed APEX Algorithm. AVPU; alert, voice, pain, unconscious

### Study population

Interrogation of our electronic clinical database HEMSbase 2.0^™^ was used to identify patients who were involved in an RTC. A search filter was applied to include terms ‘trap’, ‘extric’ or ‘pin’ to capture patients who were reportedly entrapped during either (a) the emergency call or (b) scene update prior to HEMS arrival. Patients were excluded if upon subsequent notes review the authors (EVH/JM) deemed the patient had not been trapped. Patients that were medically trapped (unable to mobilise due to the nature of their injuries rather than being mechanically trapped) were excluded, as expedited extrication is always warranted for these patients and not prohibited by the environment.

**Data Acquisition** The following patient and injury characteristics were retrieved from the electronic patient record in order to investigate the relation with the need for rapid extrication: HEMS response time, demographics, mechanism of injury, injured body regions and presenting physiology (airway status, circulation and AVPU on primary exam). Vital signs such as oxygen saturation blood pressure and (non-invasive) capnography were not considered as potential variables for the algorithm, as these require monitoring to be attached, which is sometimes not feasible and potentially first, precludes rapid decision making.

Outcome variables were collected: HEMS interventions performed on scene, pre-hospital emergency anaesthesia (PHEA), transfusion of pre-hospital blood products, surgical thoracostomies, resuscitative thoracotomy (RT) and resuscitative hysterotomy, and the hospital that the patient was conveyed too.

### Ethical considerations

This project was registered with the University of Surrey and met National Institute for Healthcare Research (NIHR, UK) criteria as a service evaluation. All the data used for this study were routinely collected as part of standard pre-hospital and in-hospital patient data collection. The project was approved by the KSS Research and Innovation Committee and conducted in accordance with Strengthening the Reporting of Observational Studies in Epidemiology (STROBE) Guidelines [12].

### Statistical analysis

Descriptive statistics are given as mean [95% CI] or median [IQR]. Baseline characteristics were compared across the subgroups using Fisher exact, Chi Square or Kruskal–Wallis tests where appropriate. To explore if certain characteristics were associated with HEMS interventions being performed after extrication, univariate correlation analysis was performed. Only variables that would be easily accessible on scene and did not require monitoring to be established were considered. Multivariable logistic regression analyses were carried out to determine which factors with an r > .2 were independently associated with the need for HEMS interventions and odds ratios (OR) were calculated for these factors. Based on the identified predictive factors, a practical algorithm was constructed. Missing values are reported in the results section of the manuscript according to the STROBE guideline. A *p*-value < 0.05 was regarded as statistically significant. All statistical analyses were conducted using SPSS 27.0 for Mac statistical package.

## Results

### Derivation of the population

During the study period, KSS attended 12,931 patients. Interrogation of the electronic patient records yielded 2859 patients meeting the pre-established search criteria for entrapment. After patient record review, 1939 patients had to be excluded as they were either not trapped (n = 1776) or medically trapped (n = 163). Subsequent results pertain to the remaining 920 physically trapped patients.

### Patient characteristics

Patient characteristics of the study population are shown in Table 1. Most patients were male (65%) and were classified as a car driver or passenger (75%). Overall, a notable proportion received HEMS interventions (29%): 124 patients (13%) received PHEA, 120 patients (13%) received a blood product transfusion, 140 patients (15%) received (unilateral or bilateral) thoracostomies and 1 patient received a resuscitative thoracotomy (RT).

**Table 1:** Patient characteristics stratified by presenting level of responsiveness (n = 920)

	All patients (n = 920)	Alert (n = 531)	Verbal (n = 212)	Pain (n = 59)	Unresponsive (n = 118)	*p*-value
Demographics						
Age, years (SD)	43 [4–99]	38 [26–60]	43 [40–46]	41 [36–46]	35 [23–54]	0.205
Male (n [%])	598 [65]	327 [62]	139 [66]	44 [75]	85 [72]	0.04
**Incident Timings**						
999 to scene time (mins)	35 [26–49]	37 [28–45]	34 [18–34]	39 [66]	32 [23–44]	0.213
Time to PHEA (hh: mm: ss)	01:11:00	01:17:00	01:16:00	01:07:00	01:06:00	0.461
**Mechanism Descriptor, n [%]**						
Car driver (or passenger)	730	424 [58]	166 [78]	45 [76]	85 [11]	0.007
Van driver (or passenger)	68	41 [60]	11 [5]	1 [2]	4 [6]	
Pedestrian*	44	25 [57]	8 [4]	2 [3]	8 [18]	
Unspecified**	78	41 [53]	27 [13]	11 [19]	21 [27]	
**Injury Descriptors, n [%]**						
Head	157 [17]	49 [9]	47 [22]	26 [44]	49 [42]	< 0.001
Chest	440 [48]	234 [44]	93 [44]	29 [49]	74 [63]	< 0.001
Abdomen	401 [43]	212 [40]	115 [54]	23 [39]	43 [36]	0.006
**Presenting Physiology, n [%]**						
**Airway**						
Obstructed Partial obstruction Managed Maintaining own *Missing*	34 55 66 753 12	0 [0] 1 [1] 0 [0] 530 [99]	0 [0] 3 [1] 1 [0.5] 207 [98]	0 [0] 31 [53] 13 [22] 15 [25]	34 [100] 18 [33] 52 [79] 2 [0.2]	< 0.001
**Circulation**						
Cardiac arrest Central pulse Radial pulse *Missing*	84 55 784 12	0 [0] 10 [2] 521 [66]	0 [0] 26 [12] 188 [89]	0 [0] 10 [17] 49 [83]	84 [68] 10 [8] 12 [10]	< 0.001
HEMS Interventions, n [%]***						
HEMS Interventions (any)	262 [29]	48 [19]	70 [27]	54 [21]	86 [34]	< 0.001
PHEA	124 [13]	16 [3]	45 [36]	48 [39]	16 [14]	< 0.001
Blood product transfusion	120 [13]	15 [3]	48 [40]	16 [13]	42 [36]	< 0.001
Thoracostomy/ies	140 [15]	17 [3]	27 [19]	23 [16]	67 [57]	< 0.001
Resuscitative Thoracotomy	1 [< 1]	0 [0]	0 [0]	1 [100]	0 [0]	0.311
**Patient Disposition and Outcome, n [%]**						
HEMS Conveyed	528 [57]	248 [47]	107 [20]	53 [10]	38 [32]	< 0.001
Pronounced Life Extinct	82 [9]	2 [< 1]	3 [4]	5 [6]	73 [62]	

Categorical data are reported as frequency (n) and percentage (%) and numerical data as median (IQR) or mean (SD). HEMS, Helicopter Emergency Medical Service; PHEA, Pre-hospital Emergency Anaesthesia; ISS, Injury Severity Score; TCA, Traumatic Cardiac Arrest *pedestrians includes those involved in an RTC who were trapped under (parts) of, or by a vehicle. **unspecified includes patients for which it was mentioned in the notes that they were trapped but who were unable to code into another category. ***CPR and associated interventions are not included under HEMS interventions as this is commonly provided by ground EMS, and HEMS activation is only considered in certain conditions.

Most patients were alert upon arrival of the HEMS team (n = 531), whereas 271 patients had a reduced level of consciousness and only responded to verbal stimuli (n = 212) or pain (n = 59). A total of 118 patients were unresponsive upon arrival.

### Provision of HEMS interventions stratified by presenting AVPU

#### Alert

HEMS interventions post-extrication (one or more) were rare in patients who were ‘alert’ on presentation: PHEA (3%), thoracostomies (3%) and blood product transfusion (3%). On electronic notes review, patients who did receive HEMS interventions despite scoring an “A” on the AVPU were characterised by a shorter than average (29 vs. 37 min) 999/112 to HEMS on-scene arrival interval, indicating that there is still potential for physiological deterioration if HEMS arrive early to scene.

#### Verbal

33% of all patients responding to voice required one or more HEMS interventions after extrication. Univariate correlation analysis (Table 2) revealed that within this group a weak but significant association was present between the absence of a fully patent airway and the need for one or more HEMS interventions post-extrication (r = .25, *p* < .001). A similar association was found for the absence of palpable radial pulses (r = .28, *p* = .08). Of the patients who responded to voice and who also had a patent airway and a palpable radial pulse, only 17% needed HEMS interventions after extrication. Of the injury descriptors, only abdominal injuries showed a weak univariate correlation with the need for HEMS interventions (Table 2). In multivariate logistic regression analysis absence of patient airway OR 6.98 [1.74–28.03] < 0.001 and absence of a radial pulse OR 9.99 [2.48–40.18] < 0.001 remained independently associated with the need for one or more HEMS interventions after extrication. Injury descriptors were no longer independently associated when entered in the same model as physiological parameters.

#### Pain and unresponsive

53/59 (90%) of the patients responding only to pain and 102/118 (86%) unresponsive patients required HEMS interventions. Patients who were unresponsive were often in (traumatic) cardiac arrest (84/118, 68%). Many received thoracostomies post-extrication (n = 67, 57%) and 36% received blood products (n = 42). Of those who were not in cardiac arrest, 16/78 (20%) received PHEA. 102/118 (86%) patients were either in cardiac arrest or received one or more HEMS interventions, the majority of these patients were pronounced life extinct (PLE) on scene but 12 were subsequently conveyed to hospital.

**Table 2:** Univariate correlation analysis for HEMS intervention performed on patients presenting with GCS 10–14 (n = 212)

Pre-hospital patient and/or treatment factor	*r*	*p-value*
Gender	0.005	0.941
Age > 65 years	0.052	0.452
Age > 80 years	0.082	0.236
Mechanism descriptor	0.093	0.283
Absent patent airway (y/n)	0.246	< 0.001**
Absent radial pulse (y/n)	0.280	< 0.001**
Chest injury (y/n)	0.103	0.136
Head injury (y/n)	0.104	0.131
Abdomen injury (y/n)	0.200	0.003*

GCS, Glasgow Coma Score; V, Voice; P, Pain. *p-value < 0.05

### Derivation of a data-driven extrication algorithm

Combining our findings and integrating these with data from previous work [8, 13] and expert opinion of best practice regarding spinal precautions with limitation of movement in the context of a patient with potential spinal injuries, has resulted in a data driven algorithm for clinical prioritisation of traumatically injured patients requiring extrication after an RTC (Fig. 1). Prior to using this algorithm the scene must be determined as safe to approach as with any standard EMS approach. The algorithm can be remembered by the acronym APEX, which stands for: AVPU score, Pulse and Airway, Evaluation of injuries and eXtrication plan “APEX”. Subsequently, the aim is to determine the level of consciousness, when the patient only responds to pain or is unresponsive, there is a high likelihood HEMS interventions are needed urgently, and rapid extrication is indicated. Simple immediate interventions (such as applying pressure to significant bleeding) should be initiated simultaneously with rapid extrication. When the patient is alert or responsive to voice, the radial pulse and airway should be evaluated. When the radial pulse is absent and/or the airway is non-patent without adjuncts, rapid extrication is warranted based on an increased likelihood of the need for HEMS interventions to be performed. When there are no obvious time critical injuries (such as major external haemorrhage), the patient has a radial pulse and a patent airway, a more controlled but timely approach for extrication with minimal patient movement can be advocated whilst continuously monitoring the patient. It is likely the patient will have sustained an injury, even if minor, during the RTC. It is possible these injuries are not immediately obvious, and the patient may have suffered a degree of bleeding which has settled with clot formation. In assisting with patient extrication, minimising movement with careful patient handling is beneficial in reducing clot disruption and limiting spinal movement. If the patient is alert, and if there is no specific concern for spinal injuries and also no pain limiting the ability to extricate, self-extrication can be used as the primary method of extrication.

## Discussion

This study has devised an algorithm based on easily obtainable variables. The data-driven algorithm may be remembered by the acronym “APEX” and can help emergency service providers on scene to determine the preferred method of extrication for patients who are trapped after a road traffic collision. This has the potential to decrease entrapment time and may contribute to a better outcome for these patients.

The proposed algorithm has several strengths. First, it is derived from real data. Unlike other extrication algorithms (i.e. for self-extrication), this algorithm was not derived in a Delphi procedure and/or based on expert-opinion, but variables included were derived from a real patient population of 920 trauma patients. Second, variables included are easy to measure, and limited clinical knowledge is required to interpret the algorithm. The algorithm will support healthcare providers in their decision on whether expedited or controlled extrication is warranted for patients entrapped in an RTC.

The study revealed that patients who were “alert” upon arrival of the HEMS team were unlikely to receive HEMS interventions post-extrication. These patients are generally not time-critical and can inform medical personnel reliably about any neurological deficits [3, 8]. Hence, a controlled extrication strategy or even self-extrication should normally be advocated in these patients. It should be noted however, that especially when these patients are seen early after their moment of injury there still is a potential for deterioration, so repeated evaluation and, if necessary, amendment of the extrication plan is warranted, as 3% of these patients still require HEMS interventions early after extrication.

The algorithm derived is most useful for patients responding to “voice”. This is a heterogenous group of patients, some of whom will require HEMS interventions and others not. In these patients, the presence of a (partially) occluded airway and absence of a radial pulse should be independently regarded as evidence for the need for expedited extrication. Extrication is required to perform critical interventions such as PHEA and blood product transfusion, and to facilitate expedited transport to definitive care (26). In support of this, evidence highlights that increased mortality is associated with a longer scene time [14, 15]. The need for spinal precautions for this group of patients should be weighed against the presence of the features mentioned above. When there are neurological deficits and when the airway is patent, and radial pulse is present, a controlled extrication technique may be preferred, whereas otherwise expedited extrication may be the approach of choice. It should be noted though that if a controlled extrication technique is chosen for this group, frequent clinical re-evaluation is mandatory, as even when they have a patent airway and a radial pulse, one or more HEMS interventions post-extrication are needed in 17% of the patients.

Patients who were only responsive to pain or were “unresponsive” almost invariably needed cardiopulmonary resuscitation and/or HEMS interventions. For these patients, rapid extrication is commonly warranted. However, sometimes to prevent the patient from dying before extrication is completed, immediate interventions whilst still entrapped are needed, such as opening the airway, gaining IV access, and starting a blood product transfusion. Whether or not these interventions should take place remains largely a dynamic risk assessment, wherein the risk of withholding these should be weighed between the risk associated with the medical professional offering these interventions, and the risk of prolonging the time to extrication. Further, the majority of unresponsive patients had a cardiac arrest and for these patients the need for rapid extrication is obvious. However, the merit of the algorithm for unresponsive patients is twofold: [1] It stresses the importance of rapid extrication of these patients when not in CA (29% of unresponsive patients), and [2] It emphasizes that for these patients sometimes interventions are needed before extrication.

The APEX algorithm allows for early decision making about the preferred extrication strategy before critical care teams such as HEMS arrive on scene. As previous studies have demonstrated entrapment after an RTC is associated with an increased mortality, decreasing entrapment time may affect outcome in this patient cohort, by shortening the time from the accident until critical (HEMS) interventions can take place. The authors hope the APEX algorithm will support those early on scene to make safe, clinically driven, evidenced based decisions for extrication and improve collaborative working between all emergency service agencies in working towards better outcomes for patients involved in RTCs. Pre-hospital systems are currently exploring the use of novel live-streaming technologies from scene using bystanders’ mobile devices [16]. These technologies can be used alongside the algorithm to ensure input from a Critical Care Dispatcher and/or Critical Care Paramedic who may be able to clinically guide the extrication in order to further improve outcome for these patients.

### Limitations

Our study has several limitations. First, although the APEX algorithm is data-driven, some aspects still rely on common sense and expert opinion: no data will be available to support recommendations on patients experiencing neurological symptoms or patients with obvious critical injuries decision making follows common sense in the instances. Second, generalisability of our study findings is potentially compromised by the fact that data were collected from one HEMS only and no data were collected from incidents with entrapment where no HEMS team attended. Third, population composition, incident types and EMS systems may differ from region to region, which may limit the external validity of the study’s findings. Further, extrication timestamps are not included on the electronic patient clinical record and therefore the relation between entrapment time and outcome could not be investigated. Finally, although the study has derived an algorithm to support decision making for extrication of trapped critically injured patients, the study has not validated the effectiveness of this algorithm in prospective practice. Therefore, multiagency services research is needed to evaluate the implementation and impact of the proposed algorithm.

## Conclusion

A simple data-driven algorithm remembered by the acronym “APEX” may help emergency service providers on scene to determine the clinically indicated and most appropriate method of extrication for patients who are trapped after a road traffic collision. This has the potential to decrease entrapment time and may contribute to a better outcome for these patients.

### Electronic supplementary material

Below is the link to the electronic supplementary material.

Supplementary Material 1

## Data Availability

The datasets used and/or analysed during the current study are available from the corresponding author on reasonable request.

## List of Abbreviations

AVPU: Alert, Voice, Pain, Unresponsive
KSS: Air Ambulance Charity Kent Surrey Sussex
ED: Emergency Department
GCS: Glasgow Coma Score
HEMS: Helicopter Emergency Medical Service
MOI: Mechanism of Injury
MTC: Major Trauma Centre
NIHR: National Institute for Healthcare Research
OR: Odds Ratio
PHEA: Prehospital Emergency Anaesthesia
RTC: Road Traffic Collision
SOP: Standard Operating Procedure
ISS: Injury Severity Score
STROBE: Strengthening of Reporting in Observational Studies in Epidemiology
EMS: Emergency Medical Services
