# Supplementary material for: A data-driven algorithm to support the clinical decision-making of patient extrication following a road traffic collision
Source: Scand J Trauma Resusc Emerg Med. 2023 Dec 4;31:90. doi: 10.1186/s13049-023-01153-2 (PMC10696863; doi:10.1186/s13049-023-01153-2)
Supplement: Supplementary file 1 — Supplementary Material 1 [file 13049_2023_1153_MOESM1_ESM.docx]

**
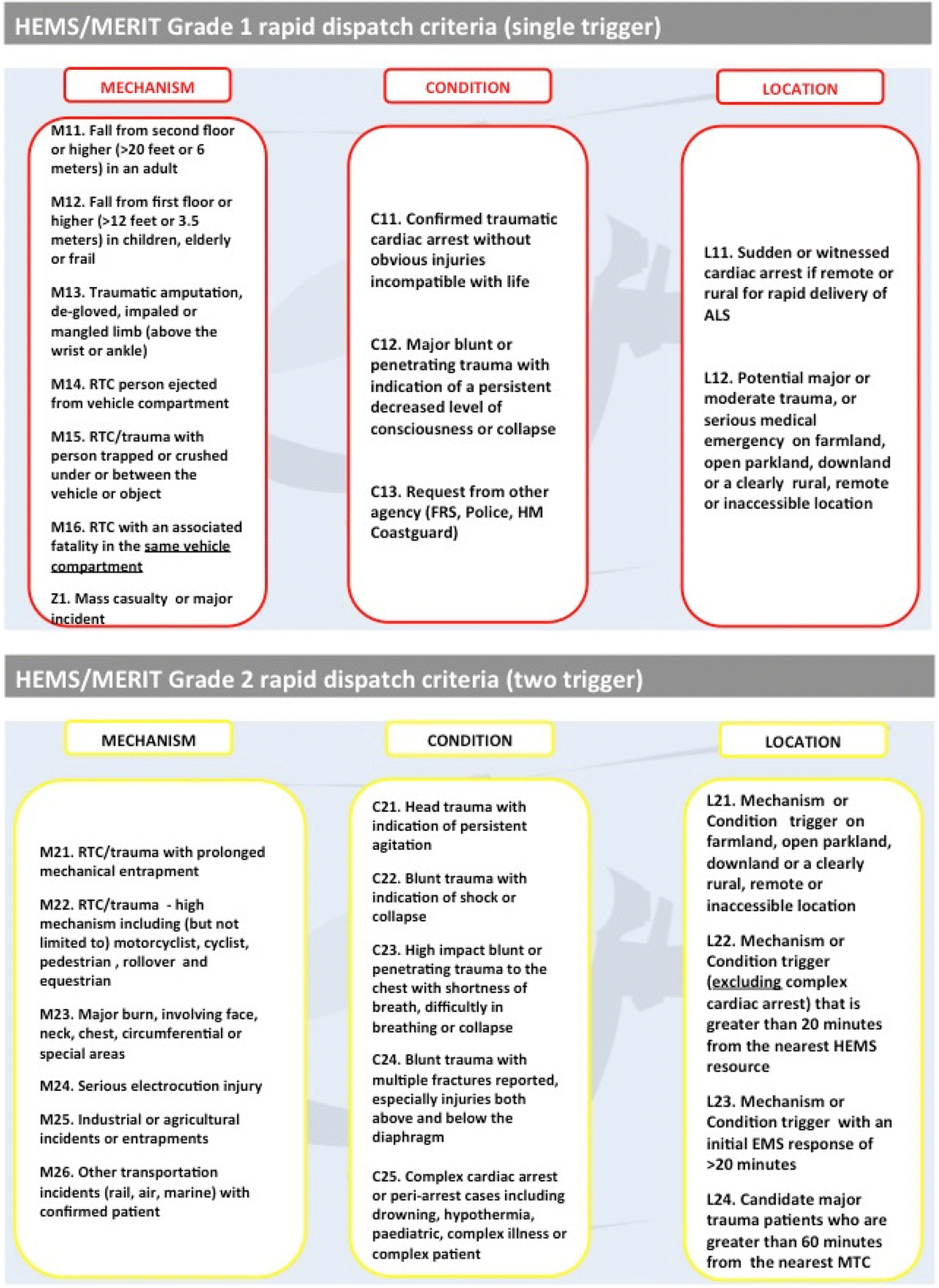
Supplementary Material Figure 1.** Tasking algorithm for Grade 1 and Grade 2 activation. Cited in Munro et al. (2018).
